# A Comparison between Several Response Surface Methodology Designs and a Neural Network Model to Optimise the Oxidation Conditions of a Lignocellulosic Blend

**DOI:** 10.3390/biom10050787

**Published:** 2020-05-19

**Authors:** Roberto López, Camino Fernández, Fernando J. Pereira, Ana Díez, Jorge Cara, Olegario Martínez, Marta E. Sánchez

**Affiliations:** 1 Physical Chemistry Area, Universidad de León, Campus de Vegazana, 24071 León, Spain; 2 Chemical Engineering Area, Universidad de León, Campus de Vegazana, 24071 León, Spain; cferrd@unileon.es (C.F.); jcarj@unileon.es (J.C.); omarm@unileon.es (O.M.); mesanm@unileon.es (M.E.S.); 3 Analytical Chemistry Area, Universidad de León, Campus de Vegazana, 24071 León, Spain; fjperg@unileon.es; 4 Electrical Engineering Area, Universidad de León, Campus de Vegazana, 24071 León, Spain; ana.diez.suarez@unileon.es

**Keywords:** oxy-combustion, biomass, factorial design, Monte Carlo, optimisation, neural network

## Abstract

In this paper, response surface methodology (RSM) designs and an artificial neural network (ANN) are used to obtain the optimal conditions for the oxy-combustion of a corn–rape blend. The ignition temperature (*T_e_*) and burnout index (*D_f_*) were selected as the responses to be optimised, while the CO_2_/O_2_ molar ratio, the total flow, and the proportion of rape in the blend were chosen as the influencing factors. For the RSM designs, complete, Box–Behnken, and central composite designs were performed to assess the experimental results. By applying the RSM, it was found that the principal effects of the three factors were statistically significant to compute both responses. Only the interactions of the factors on *D_f_* were successfully described by the Box–Behnken model, while the complete design model was adequate to describe such interactions on both responses. The central composite design was found to be inadequate to describe the factor interactions. Nevertheless, the three methods predicted the optimal conditions properly, due to the cancellation of net positive and negative errors in the mathematical adjustment. The ANN presented the highest regression coefficient of all methods tested and needed only 20 experiments to reach the best predictions, compared with the 32 experiments needed by the best RSM method. Hence, the ANN was found to be the most efficient model, in terms of good prediction ability and a low resource requirement. Finally, the optimum point was found to be a CO_2_/O_2_ molar ratio of 3.3, a total flow of 108 mL/min, and 61% of rape in the biomass blend.

## 1. Introduction

Throughout the world, particularly in developed countries with high populations (e.g., Mexico, Brazil, India, and China) and those countries which consume the most energy per capita (e.g., Iceland, Canada, the U.S., and so on) [1,2], there has been a continuous increase in demand for energy production. This demand is partially supported by the use of clean energies but is still also supported by the combustion of fossil fuels. Many authors have identified the principal cause of this increase in energy demand to be economic development and population growth. The International Energy Agency (IEA) expects a continuous rise of energy demand in Organisation for Economic Co-operation and Development (OECD) countries. Under current and planned policies, as modelled by the IEA in the New Policies Scenario, energy demand is set to grow by more than 25% by 2040, requiring an investment of more than $2 trillion a year into new energy supply [3]. At the same time, the global energy demand is predicted to grow by more than a quarter between 2018 and 2040. On the other hand, geopolitical uncertainties in the Middle East have established increasing concerns about the future of oil supplies. In Europe, the recent decision of the United Kingdom to leave the European Union has no precedent in European uncertainties [4]. Regarding pollutant emissions, the United Nations Climate Change Annual Report published in December 2018 pointed out that, on the basis of the current trends of greenhouse gas emissions, the global average temperature will increase by 3–5 °C by 2100. In addition, extreme weather events already account for 91% of all major disasters and 77% of recorded economic losses from natural disasters [5]. To avoid this situation, greenhouse gas emissions—mainly produced by the combustion of fossil fuels—must be dramatically reduced in the coming years [6,7,8]. In the same direction, the European Union (EU) has planned a linear reduction factor of 2.2% for CO_2_ emissions over the next decade (i.e., 2021–2030) [9].

Alternative technologies to energy production based on the combustion of fossil fuels can be classified into two main groups: those with and without CO_2_ capture. In the first group, also called Carbon Capture and Storage (CCS) technology, the oxy-combustion of fuels such as coal or biomass is a very interesting strategy to reduce the environmental impact of actual coal-fired power plants, as it is possible to obtain a very rich output flow of CO_2_—usually at more than 90% concentration—which can be stored without important greenhouse gas emissions [6,10,11]. The use of biomass as oxy-combustion feedstock is one way to use the solar power radiation of the atmosphere, which is estimated to be over 10^17^ W. Plants only collect and use 0.02% of this; however, they still produce a total annual energy storage of 10^21^ J [12]. The use of biomass as feedstock in oxy-combustion power plants has successfully been proposed and widely studied in previous studies [13,14,15]. To date, however, there has been little discussion about using biomass blends as the only raw material in oxy-combustion processes for the production of electricity [16,17]. To improve the profitability of thermal processes, optimisation techniques play a very important role in engineering designs. Thus, optimisation methods have evolved from the most simple mathematical regressions to the most advanced artificial intelligence (AI) methods [18,19]. Despite the variety of available optimisation methods, they all share a need for knowing the critical parameters which affect the variable that is optimised. For example, in oxy-combustion processes, the presence of char in the feedstock and the volatile compounds produced in oxy-combustion or flame ignition are factors which must be considered [20]. The atmospheric composition (i.e., the amount of CO_2_ or the presence of SO_2_), particle sizes, and the reactor flow are the main external and manipulated variables in the literature [11,21,22].

With the aim of evaluating the application of different optimisation methods for the oxy-combustion of a biomass blend, three response surface methodology (RSM) techniques and an artificial neural network (ANN) are applied in this work. The experimental data necessary to obtain these models were obtained in our laboratories and we collected the corn and rape used as biomass from planting zones around the city of León (Spain). With the development of AI, the ANN has shown great potential as an effective tool for handling complex non-linear problems. These methods have been applied to very different contexts, such as the mining industry, metallurgy, enterprise organisation, or building [23,24], as well as, in particular, to the optimisation of chemical processes [25,26,27,28,29]. On the other hand, RSM methods belong to more classical methods, but present high efficiency in optimisation tasks. Consequently, in this paper, ANN optimisation was compared with optimisation by the following RSM methods: a complete design, a Box–Behnken design, and a central composite design [25,30,31,32,33,34,35,36,37]. For the sake of comparison, the point of best operability was reached when a simultaneous minimum ignition temperature and a maximum burnout index were achieved. These two optimal points were chosen because they are related with the most effective factors in the process: a lower ignition temperature (*T_e_*) is related with a reduction of the energy needs and, consequently, the cost of the process. On the other hand, a high burnout index (*D_f_*) represents better oxy-combustion of the biomass and, thus, less final residue. The influence of the atmosphere (CO_2_/O_2_ ratio), the total flow, and the proportion of rape in a corn–rape blend on the oxy-combustion were selected as the main factors to be optimised in the experimental design. Finally, for the sake of validation, the results obtained with the RSM model with the best adjusting parameters were compared with those obtained by a Monte Carlo simulation. The conclusions obtained by the evaluation of the proposed scenarios add to the growing body of literature on oxy-combustion technology and are considered useful to improve several alternative processes to traditional electric production (i.e., by only coal combustion). For the sake of clarity, the main aim of this work was to obtain the optimal conditions to oxidise a corn–rape blend. However, as RSM are considered in the literature to be “traditional” (or well-known) procedures, while ANN methods have not been explored in many research fields, we also asked whether the potential of ANN methods is high enough for them to supersede RSM methods in optimisation research.

## 2. Materials and Methods

### 2.1. Samples

The untreated biomass used in this study was taken from the branch and leaf wastes of corn and rape plants in the region of León, Spain. The bioresidues were dried, crushed, and sieved under 90 µm [38]. Before the thermogravimetric analysis, the bioresidues were characterised to determine their proximate and ultimate characteristics, as well as their corresponding heating values. Moisture content was determined gravimetrically using the oven drying method (D4442). The higher heating value (HHV, D240) at a constant volume was measured by an adiabatic oxygen bomb calorimeter C-6000 AZOM, London, UK (sourced from Spain). Proximate determinations were made in accordance with modified procedures from E870 (Standard Methods for Analysis of Wood Bioresidues), D1102 (ash in wood), and E872 (volatile matter). For the ultimate analysis, a LECO CHN-600 instrument, Berlin, Germany (sourced from Spain) was used to determine the carbon, hydrogen, and nitrogen content (D5373). Sulphur was analysed using a LECO SC-132 instrument, Berlin, Germany (sourced from Spain) (D4239). As corn and rape are lignocellulosic bioresidues, the cellulose, hemicellulose, and lignin compositions of the bioresidues were determined by duplicate analyses of neutral detergent fibre (NDF), acid detergent fibre (ADF), and crude fibre [39] in ground samples using an Ankom 200 fibre analyser, New York, NY, USA (sourced from Spain). A proximate analysis was also taken into account [6] for the discussion of the results. The results of the proximate and ultimate analyses, calorific values, and composition of principal lignocellulose components corresponding to the corn and rape are listed in Table 1.

### 2.2. Empirical Models: Factorial Design and the Desirability Function

RSM is a statistical technique which is experimentally used to study the relationships between an objective variable (also called the response) and diverse decision variables (also called factors), in order to determine the optimal experimental conditions for the desired response [37,40,41,42]. In this study, firstly, the response (y_i_) and the factors (x_i_) were fitted to a first-order polynomial. If the regression was not linear, then a second-order polynomial was attempted. Once this regression was considered appropriate to describe the response–factor relationships, it was necessary to determine whether a maximum, a minimum, or a saddle point had been found by calculating the eigenvalues of the matrix formed by the second-order coefficients of the quadratic model. Finally, the optimal point (also called the stationary point) was obtained by solving the equation system obtained from the partial derivative of the response with respect to each independent variable [37].

The approximation for the second-order model of a three-factorial design can be written as

(1) $$y=\beta_{0}+\beta_{1}x_{1}+\beta_{2}x_{2}+\beta_{3}x_{3}+\beta_{12}x_{1}x_{2}+\beta_{13}x_{1}x_{3}+\beta_{23}x_{2}x_{3}+\beta_{11}x_{1}^{2}+\beta_{22}x_{2}^{2}+\beta_{33}x_{3}^{2}.$$

This second-order equation can be written, in matrix form, as

(2) $$\hat{Y}=\beta_{0}+x’b+x’Bx,$$

where the vector **b** contains the coefficients of the linear expression (principal effects) and the matrix B is formed by coefficients which represent interactions and pure quadratic terms [40].

The optimal point is calculated by solving the following expression:

(3) $$\frac{\partial \hat{Y}}{\partial x}=b+2Bx=0,$$

and, hence, the optimal point is obtained as

(4) $$x_{s}=-\left(\frac{1}{2}\right)B^{-1}b.$$

The signs of the eigenvalues (*λ_i_*) of the matrix B determine whether the stationary point is a maximum, a minimum, or a saddle point. If all the signs of the eigenvalues of B are positive, the stationary point corresponds to a minimum. If all the signs of the eigenvalues are negative, the stationary point corresponds to a maximum. Finally, if the signs of eigenvalues are both positive and negative, a saddle point must be attributed to the stationary point [37,40]. For the sake of optimisation, a desirability function was used and all the responses were combined into one measurement [32]. As a result, the optimal levels of the factors were obtained. To achieve the mathematical treatment, the Design-Expert^®^ v.11 software was used [43].

Combination of the responses into one desirability function requires the calculation of each individual desirability function [44]. This method finds the operating conditions (*x)* that provide the “most desirable” response values. For each response *Y_i_ (x)*, a desirability function, *d_i_ (Y_i_)*, assigns numbers between 0 and 1 to the possible values of *Y_i_*, where *d_i_ (Y_i_)* = 0 represents a completely undesirable value of *Y_i_ (x)* and *d_i_ (Y_i_)* = 1 represents a completely desirable (or ideal) response value. Hence, if a response must be maximised, the individual desirability is defined as

(5) $$di\;\left(Yi\right)=\left\{\begin{array}{c} 0\;\;\;\;\;\;\;\;\;\;\;\;\;\;\;\;\;\;\;\;\;\;\;\;\;\;\;\;if\;\;\;\;\;\;\;Y_{i}<Y_{min}\;\\ \frac{Y_{i}-Y_{min}}{Y_{max}-Y_{min}}\;\;\;\;\;\;\;\;\;\;\;\;\;\;\;if\;\;\;Y_{min}<Y_{i}<Y_{max}\;\\ \;1\;\;\;\;\;\;\;\;\;\;\;\;\;\;\;\;\;\;\;\;\;\;\;\;\;\;\;\;if\;\;\;\;\;\;\;Y_{i}>Y_{max}\; \end{array}\right.$$

If a response must be minimised, we can use

(6) $$dj\;\left(Yi\right)=\left\{\begin{array}{c} 1\;\;\;\;\;\;\;\;\;\;\;\;\;\;\;\;\;\;\;\;\;\;\;\;\;\;\;\;if\;\;Y_{i}<Y_{min}\;\\ \frac{Y_{i}-Y_{min}}{Y_{max}-Y_{min}}\;\;\;\;\;\;\;\;\;\;\;\;\;\;\;\;if\;\;\;Y_{max}<Y_{i}<Y_{min}\;\\ 0\;\;\;\;\;\;\;\;\;\;\;\;\;\;\;\;\;\;\;\;\;\;\;\;\;\;\;\;if\;\;Y_{i}>Y_{max}\; \end{array}\right.$$

Finally, the overall desirability values are calculated from the individual values by using the following equation for the two responses:

(7) $$D={\left(d_{1}\;\times \;\;d_{2}\right)}^{1/2}.$$

The geometric mean shown in Equation (7) has the property that if either model is undesirable (i.e., *d_i_* or *d_j_* is zero), the overall desirability is also unacceptable (*D* = 0). Therefore, the desirability function method allows us to simultaneously optimise a series of quadratic models. A response surface experiment may use measurements on a set of outcomes. Instead of optimising each outcome separately, the settings for the predictor variables seek to satisfy all of the outcomes at once.

In this study, a 3^3^ complete factorial design was used to determine the effects of the atmosphere composition (CO_2_/O_2_ ratio) (x_1_), the total flow of the oxidising gases (x_2_), and the proportion of rape in the corn–rape blend (x_3_) on two responses: the ignition temperature (y_1_) and the burnout index (y_2_). The selection of the most important factors affecting the overall oxidation process was based on conclusions achieved in our previous studies [38,45,46,47,48]. The temperature ramp was fixed, in terms of best operational time, as studied and optimised in our first work [38]. The particle diameter was fixed in order to avoid internal diffusion, based on a previous study comparing corn, sunflower, rape, and a microalgae (*Scenedesmus almeriensis*); the microalgae was a green powder with a 90 µm average particle size [45]. In another work [47], we proved that this size was good enough to obtain a uniform distribution of temperature and concentration within the particle and limit external resistances to heat and mass transport. Consequently, we sieved all biomass under 90 µm, in order to study bioresidues with the same particle diameter. The total flow was selected as a factor to be optimised as it plays a significant role in the presence or absence of external diffusion as a control step. The CO_2_/O_2_ ratio was selected because it is very important in evaluating the extent of oxidation of the volatile matter and the char produced during the oxidation process. Finally, the proportion of rape in the blend was studied as it determines the main lignocellulosic component distribution and, hence, the existence of positive or negative synergies between them.

In addition, two reduced models were applied and compared in the study: a Box–Behnken model and a 3^2^ central composite design. Table 2 shows the differences between the three RSM methods used for optimisation.

The complete design can be situated in the limit of the experimental allowed cost. It is the most desirable experimental design when three factors are to be studied. However, the need for 32 experiments can be reduced to only 19 (or even 17) if a reduced model is applied. Therefore, these methods must be explored, in order to reduce the cost of the experimental section. The main advantage of the three models previously described is their rotatability, providing a constant prediction variance at all points which are equidistant from the design centre. However, the main disadvantage of the reduced models is worse data prediction, due to their lower input experimental values than the complete design model. Considering the reduced models, although the Box–Behnken model is less expensive to run than the central composite design (with the same number of factors), there are some experimental situations which make the selection of a Box–Behnken design impossible (e.g., when the interval of the levels of a factor causes −α to be negative for a variable that must be positive, such as the pressure) [30,43]. In this manuscript, both reduced models could be selected for the response optimisation and, therefore, both were explored.

As the last step, the accuracy of the best adjusted model of those described above was studied by comparing the optimal values reported by its application with those reported by a Monte Carlo simulation. In this simulation, 100 experiments were carried out by maintaining the CO_2_/O_2_ ratio and total flow of oxidising gases at the optimal values derived from the most accurate method. Hence, the rape proportion in the corn–rape blend was varied. After 100 experiments, both responses were correlated by obtaining a Monte Carlo (MC) index, defined as

(8) $$MC\;\left(x_{3}\right)=T_{e}\;-\;\;D_{f}.$$

Afterwards, the MC index was minimised in the set of the 100 experiments to obtain an optimal proportion of rape in the corn–rape blend. Finally, this value was compared with that obtained with the RSM.

### 2.3. Artificial Intelligence Model: the Artificial Neural Network

#### 2.3.1. Fundamentals

An ANN is a mathematical model which is trained to reproduce the structure of a biological neural network, which was discovered by Ramón and Cajal in 1888 [49], with the aim of reaching a similar functionality. The objectives to be emulated are: (1) parallel processing; (2) distributed memory by the network synapses; and (3) adaptability to the environment, which allows the model to learn from previous experiences. The basic model of an artificial neuron was introduced by Rumelhart and McClelland [50], based on the following components:

1. The input variables, *x_j_*, and their synaptic weights *w_ij_*, (*j* = 1, …, *n*).
2. A propagation rule, *h_i_*, which is based on the input variables and their synaptic weights. This can be expressed by an additional parameter, *θ_i_*, also called the bias. If we let the *i* and *j* indices begin at zero, and set *w_i0_* = *θ_i_* and *x_0_* = −1, the propagation rule can be expressed as
  (9) $$h_{i\;}(x_{1},\;\ldots ,x_{n},w_{i1},\ldots ,w_{in}=\overset{n}{\underset{j=0}{\sum }}\;w_{ij}x_{j}=\overset{n}{\underset{i=1}{\sum }}w_{ij}x_{j}-\theta_{i}.$$
3. An activation function, *p_i_*, which represents the output of a neuron and its activation state at the same time. The activation function can be obtained as
  (10) $$p_{i}=f_{i}\;\left(h_{i}\right)=f_{i}\;(\sum_{j=0}^{n}w_{ij}x_{j}).$$

To obtain a neural output from its net input, a neural transfer function, *tansig,* must be used. This function may be expressed as follows:

(11) $$p_{i}=\frac{1}{1+e^{-\left(\sum_{j=1}^{n}w_{ij}x_{j}-\theta_{i}\right)}},\;p_{i}\;\in \;\left[0,1\right].$$

The previous expression is a continuous and differentiable function, which is necessary for the application of a backpropagation neural network (BPN) based on the Multi-Layer Perceptron (MLP) developed by Rumelhart et al. [51]. This method is known to be one of the most representative learning models for the ANN [52]; which is the reason it was selected for this study.

**Figure 1:**
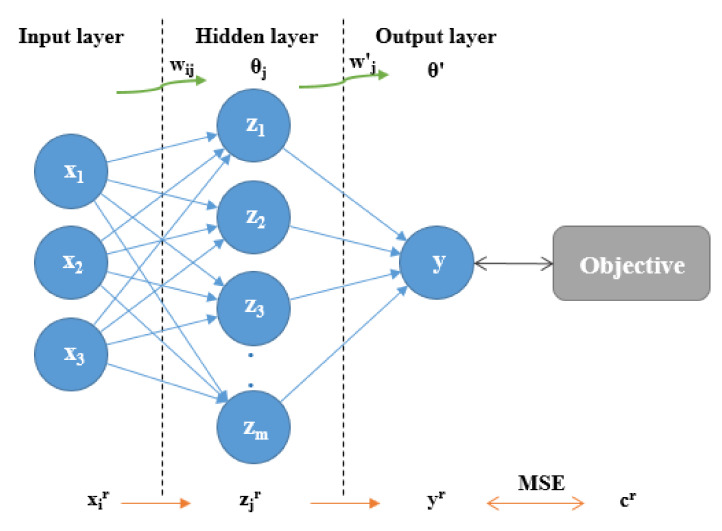
A scheme of the topological structure of the Multi-Layer Perceptron method.

The topology architecture of the ANN is critical to obtain a good model which provides the desired output for a given input. As shown in Figure 1, the MLP model is based on the existence of an input neuron layer, one or more hidden neuron layers, and an output neuron layer. For the sake of simplicity, the MLP model will be explained only considering one hidden layer and one neuron in the output layer in the ANN architecture. In this case, if *x_i_* denotes the *n* input variables, *z_j_* denotes the *o* outputs from the hidden layer and *y* denotes the output of the final layer, which may be compared with the objective output value *c*. Furthermore, we denote *w_ij_* to be the weight of the hidden layer, *θ_j_* the corresponding bias values, *w’_j_* to be the weight of the output layer, and *θ’* the respective bias. With input variables given by the vector *x^r^* (*r* = 1, …, *N*), the MLP model operates as follows:

(12) $$y^{r}=\overset{o}{\underset{j=1}{\sum }}w{´}_{j}z_{j}^{r}-\theta ´=\overset{o}{\underset{j=1}{\sum }}w{´}_{j}f\left(\overset{n}{\underset{i=1}{\sum }}w_{ji}x_{i}^{r}-\theta_{j}\right)-\theta ´.$$

To analyse the convergence of the method in successive iterations, the mean square error (MSE) is defined as

(13) $$MSE\;\left(w,\;w´,\theta ,\theta ´\right)=\frac{1}{2}\overset{N}{\underset{r=1}{\sum }}{\left(c^{r}-y^{r}\right)}^{2}.$$

For the sake of optimisation, the gradient descent algorithm is performed for the backpropagation learning technique to create a highly trained ANN [27,28]. The gradient referring to the weight of the output layer and that of the hidden layer are, respectively,

(14) $$∆w{´}_{j}=-\varepsilon \frac{\partial \left(RSM\right)}{\partial w{´}_{j}},$$

(15) $$∆w_{ji}=-\varepsilon \frac{\partial \left(RSM\right)}{\partial w_{ji}}.$$

Finally, the actualisation of the weights, in two successive iterations, are obtained as

(16) $$∆w{´}_{j}=\varepsilon \overset{N}{\underset{r=1}{\sum }}\left(c^{r}-{\left(\overset{o}{\underset{j=1}{\sum }}w{´}_{j}z_{j}^{r}-\theta ´\right)}^{2}\right)z_{j}^{r},$$

(17) $$∆w_{ji}=\varepsilon \overset{o}{\underset{j=1}{\sum }}(w{´}_{j}z_{j}^{r}-\theta ´)w{´}_{j}\frac{\partial f\left(\sum_{i=1}^{n}w_{ji}x_{i}^{r}-\theta_{j}\right)}{\partial \left(\sum_{i=1}^{n}w_{ji}x_{i}^{r}-\theta_{j}\right)}x_{i}^{r}.$$

The actualisation of the biases in two successive iterations are also obtained with Equations (16) and (17), considering that the bias is a particular case of a synaptic weight, where the input is −1. Thus, in this paper, two ANN models were used for the optimisation. In both, the inlet layer was considered to have three neurons, corresponding to the variables of CO_2_/O_2_ ratio (x_1_), the total flow of oxidising gases (x_2_), and the proportion of rape in the corn–rape blend (x_3_). On the other hand, the outlet layer was computed with one neuron in each model: the ignition temperature in ANN_1_ and the burnout index in ANN_2_. The number of hidden layers was obtained by studying the evolution of the MSE with the number of neurons for one and two hidden layers. Finally, by successive iterations based on Equations (11)–(17), the adjusted parameters of the ANN were obtained by minimising the MSE with an experimental data set (as the training data set). Afterwards, the model was validated using a validation experimental data set.

Once ANN_1_ and ANN_2_ were successfully trained and validated, they were used to optimise the variables of the oxy-combustion process. As for RSM, the variables to be optimised were also the CO_2_/O_2_ ratio (x_1_), the total flow of oxidising gases (x_2_), and the proportion of rape in the corn–rape blend (x_3_). These three variables were situated at the input layer. ANN_1_ generated the ignition temperature (*T_e_*) value for each triplet (x_1_, x_2_, x_3_) tested, while ANN_2_ calculated the burnout index (*D_f_*) for the same or another input triplet. For the sake of optimisation, a variable called the optimisation index, OI, was defined as follows:

(18) $$OI\;\left(x_{1},\;x_{2},x_{3}\right)=T_{e}\;-\;D_{f}.$$

**Figure 2:**
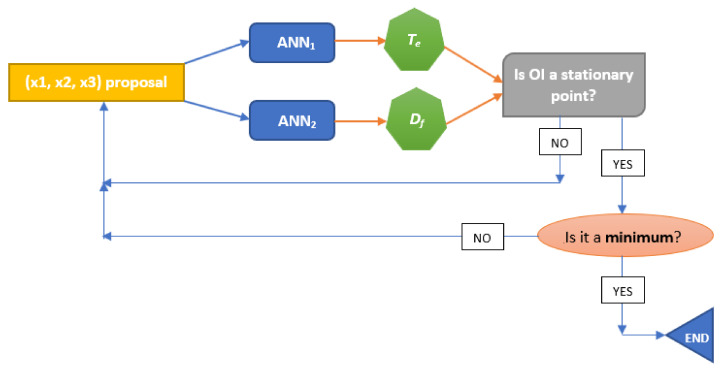
Optimisation scheme of the following iterations for artificial neural network (ANN) method. (OI: optimization index).

As the optimisation consisted of obtaining a triplet (x_1_, x_2_, x_3_) to compute a minimum value of *T_e_* and a maximum value of *D_f_*, the OI had to be minimised. Therefore, the mathematical procedure in the optimisation step consisted of computing a high number of (x_1_, x_2_, x_3_) results, in order to obtain a minimum value of OI inside the intervals proposed for the CO_2_/O_2_ ratio (x_1_), the total flow of oxidising gases (x_2_), and the proportion of rape in the corn–rape blend (x_3_). Each triplet in the first step produced an OI value. When a minimum value of all the triplet sets was obtained, the computation was finished. A scheme of this iterative procedure is presented in Figure 2.

#### 2.3.2. Training and Validation Data Sets

In this study, for the sake of economy, the training and validation data sets included all possible experiments that we had to perform in the RSM methods. The training data set was composed of 20 experiments. To ensure the generality of the proposed AI method, the following ranges were selected: CO_2_/O_2_ molar ratio between 2 and 5, total flow (mL/min) between 75 and 125, and proportion of rape in the blend between 25% and 75%. The star points of the Box–Behnken design were also included. These conditions were selected based on our previous studies [46,47], and were in accordance with the literature study performed previous to these works and cited elsewhere.

After the calculation of the parameters of the ANN, the performance of the trained model was evaluated by studying the prediction of the output variables—the ignition temperature (y_1_) and the burnout index (y_2_)—for 18 different conditions. This final step allowed us to examine the model in a comprehensive manner.

### 2.4. Data Collection

To understand how the combustion atmosphere and the chemical composition affect the biomass thermal decomposition, different oxy-combustion experimental series were performed in a SDTQ600 analyser (TA Instruments, New Castle, DE, USA) (for thermogravimetric analysis, TGA). These series were in accordance with the RSM and the ANN used in each case. Table S1 in the Supplementary Materials provides the selected values for each factor and the different formulations of each considered factorial design.

The mass loss was recorded continuously as a function of time and temperature, from room temperature to 1173 K, using a heating rate (β = dT/dt) of 20 K/min. This temperature rate and the development of the experimental setup were the same as those used in previous studies [45].

### 2.5. Determination of the Oxy-Combustion Indexes

The ignition temperature (*T_e_*) and burnout index (*D_f_*) were chosen as the objective variables to be optimised. The ignition temperature was chosen as it is a characteristic index of the beginning of the ignition and the burnout index was chosen because it is a characteristic parameter of the end of biomass oxidation.

#### 2.5.1. Ignition Temperature (T_e_)

**Figure 3:**
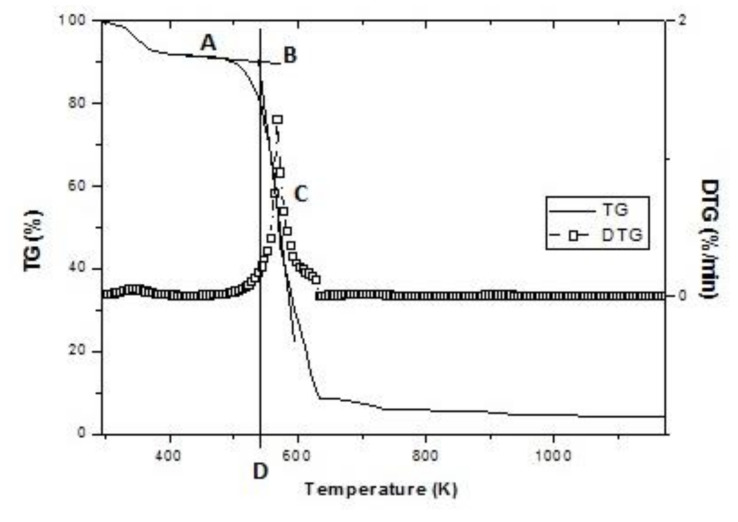
Ignition temperature (*T_e_*) definition sketch. (TG: thermogram; DTG: first derivative of the thermogram).

As described by Ma et al. [53], Nie et al. [54], defined in previous studies [45], and shown in Figure 3, the ignition temperature (*T_e_*) was defined as follows. First, through the first derivative of the thermogram (DTG )peak point, a vertical line was drawn downward to intersect the thermogram (TG) oblique line at point C. Secondly, a tangent line to the TG curve was made at point A, which intersected the extended TG initial level line at point B. Thirdly, another vertical line was drawn downwards through the point B, which met the cross axis at a point D. The temperature corresponding to the point D was defined as *T_e_*.

#### 2.5.2. Burnout Index (D_f_)

The burnout index can be described as follows [45]:

(19) $$D_{f}=\frac{{\left(\frac{dw}{dt}\right)}_{max}}{∆t_{1/2}t_{p}t_{f}}$$

where (d*w*/d*t*)_max_ is the maximum combustion rate, *Δt*_1/2_ is the time zone where (d*w*/d*t*)/(d*w*/d*t*)_max_ = 1/2, *t*_p_ is the time corresponding to (d*w*/d*t*)_max_, and *t*_f_ is the burnout time, which corresponds to the time at which two consecutive TG points are much less than 5%.

## 3. Results and Discussion

### 3.1. General Comments and Section Organisation

The results of this research are organised into three groups:

1. The RSM results (Section 3.2, Section 3.3, Section 3.4, Section 3.5, Section 3.6), which were used to understand the effect of each factor and the effect of the interactions of the factors on each response. These results include the mathematical model obtained for each RSM applied.
2. The ANN results (Section 3.7), including the optimisation of the number of hidden layers, the number of neurons of each hidden layer, and the parametrisation of the ANN model. The validation of the model is included in this section.
3. Comparison between all of the optimisation methods tested (Section 3.8).

**Figure 4:**
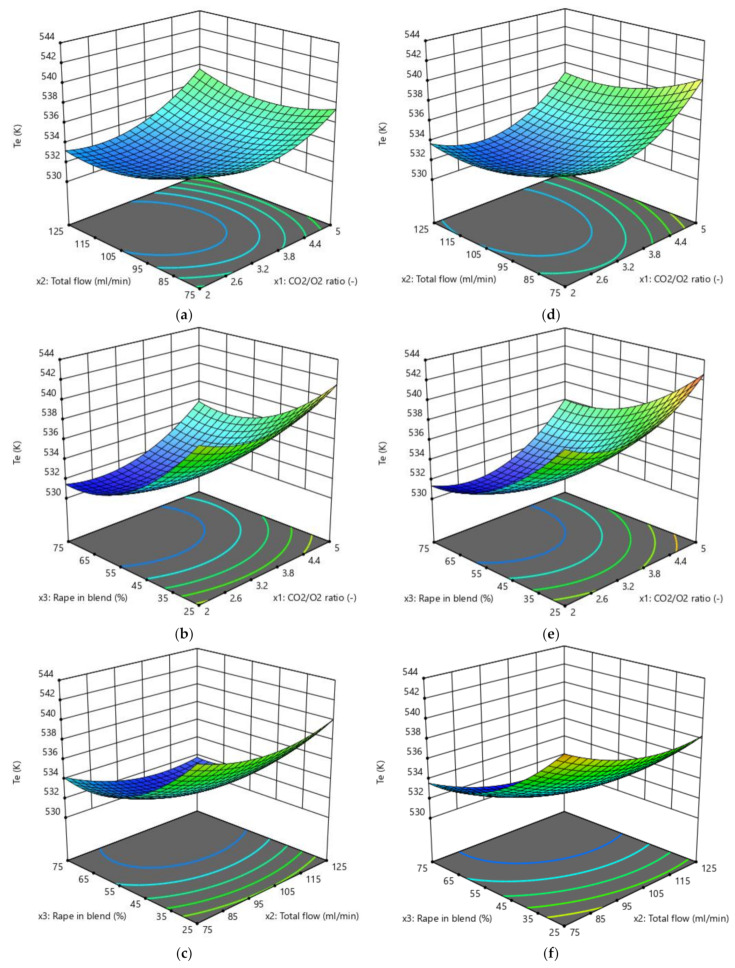
Ignition temperature (*T_e_*) response surfaces: (**a**–**c**) Complete design; and (**d**–**f**) Box–Behnken design.

**Figure 5:**
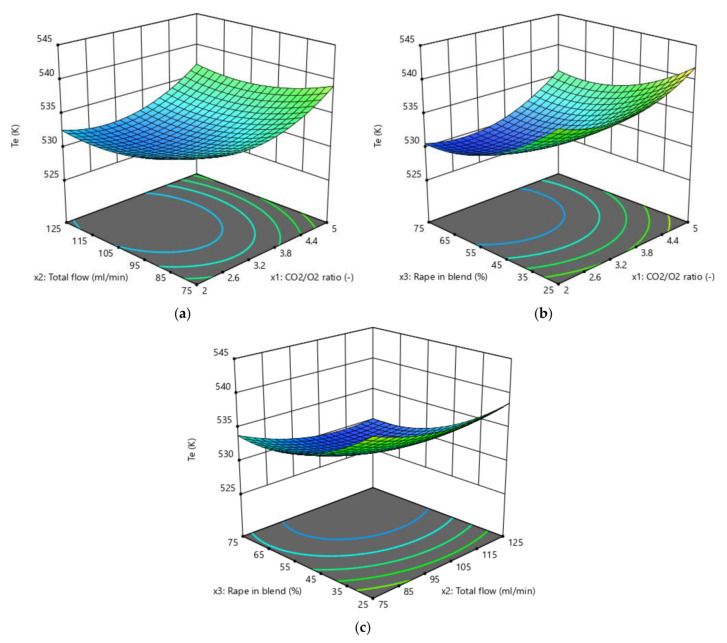
*T_e_* response surfaces for the central composite design (**a**–**c**).

**Figure 6:**
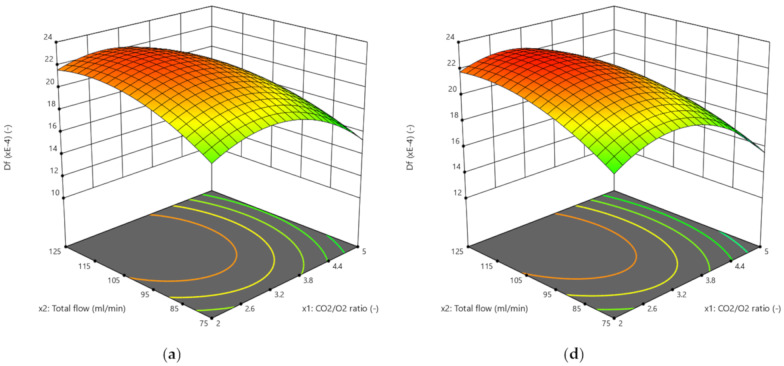

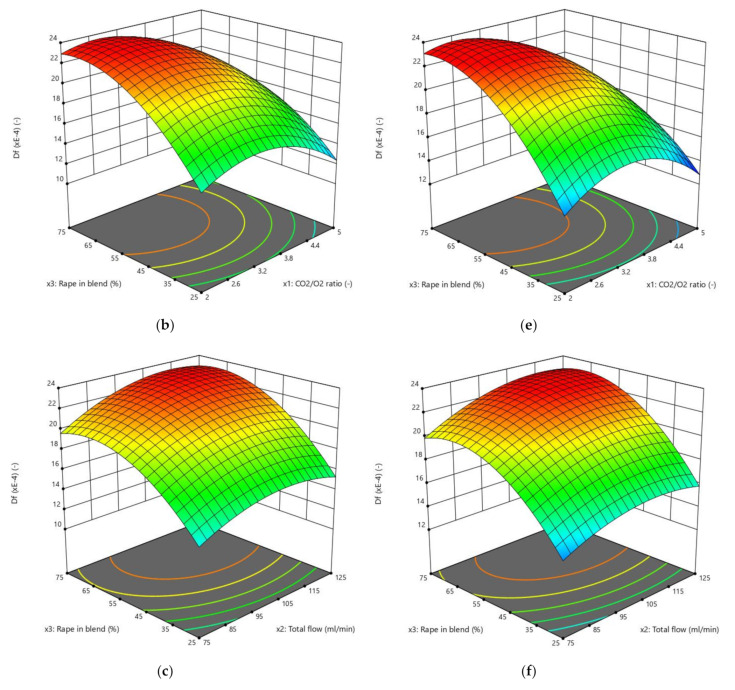
Burnout index (*D_f_*) response surfaces: (**a**–**c**) Complete design; and (**d**–**f**) Box–Behnken design.

**Figure 7:**
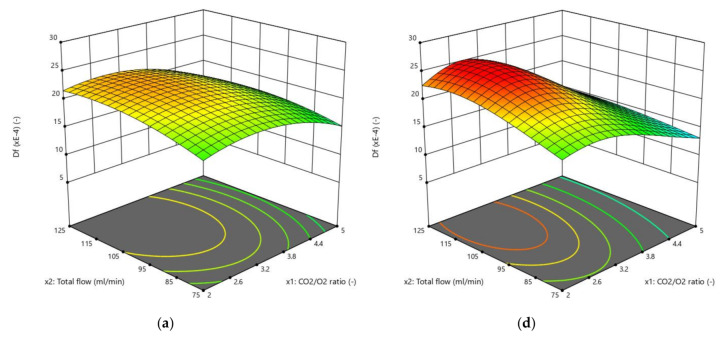

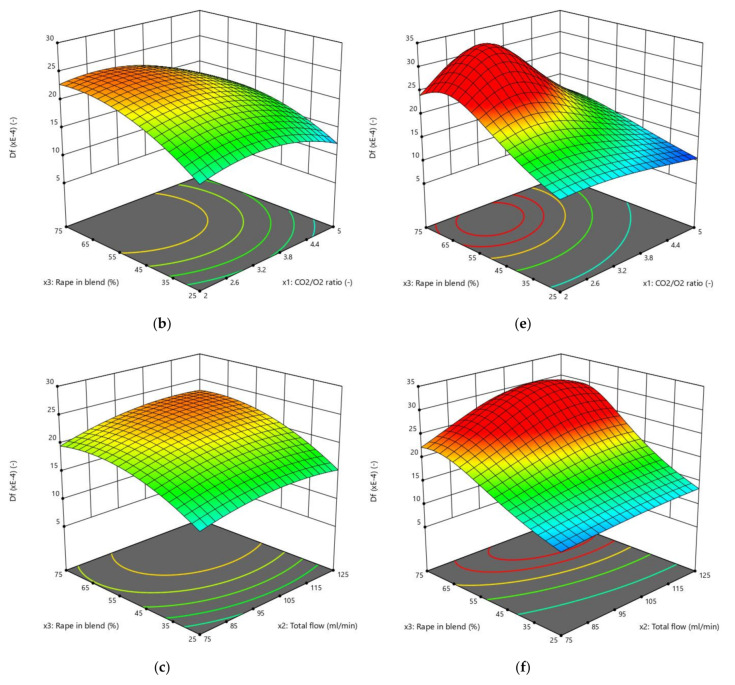
*D_f_* response surfaces: (**a**–**c**) Central composite design; and (**d**–**f**) Central composite design with power transformation.

The results obtained by the three RSM methods are presented in Figure 4, Figure 5, Figure 6 and Figure 7. The ignition temperature response is shown in Figure 4 and Figure 5, while the burnout index results are presented in Figure 6 and Figure 7. Figure 4a–c presents the response surfaces of the ignition temperature when the complete design was applied, while Figure 4d–f and Figure 5a–c presents the same surfaces when the Box–Behnken and central composite design, respectively, were used. Due to the 3D limitations, the figures had to be obtained by computing couples of factors. Therefore, Figure 4a presents the different *T_e_* values that could be obtained in the mathematical surface when the CO_2_/O_2_ ratio (x_1_) and the total flow of the oxidising gases (x_2_) varied between the limits presented in Tables S1–S3. In Figure 4b,e and Figure 5b, (x_1_) and the proportion of rape in the corn–rape blend (x_3_) are used. Finally, in Figure 4c,f and Figure 5c, (x_2_) and (x_3_) are used for the calculations. The only difference between the figure sets 4a–c, 4d–f, and 5a–c is the RSM model applied.

Figure 6 and Figure 7 present the burnout index response, which were obtained using the same procedure as for Figure 4 and Figure 5. In addition, Figure 7 also presents the results of applying a power transformation to the *D_f_* data (Figure 7d–f).

As an example, observing Figure 4b, it can be concluded that the maximum ignition temperature is obtained when the factor (x_1_) is at its highest level while, at the same time, (x_3_) is at its lowest level. It is also true that the lowest *T_e_* is reached when (x_1_) is lowest and (x_3_) is highest. This trend was similar in the three RSM models, but there were differences both in the response values and in the slopes of the surfaces. The previous analysis was done by maintaining the (x_2_) factor at the value corresponding to each design planification (see Tables S1–S3). However, when the surface presented a higher planarity (e.g., as in Figure 4f), it was more difficult to see how the response changed when one factor varied.

From an initial observation, it can be concluded that all the models presented an extremal point. From an individual point of view of the responses, this finding validates the numerical interval selected for each factor in this study (see Table S1 in the Supplementary Materials).

In addition to the previous comment, the shape of the surface was similar in all cases. The shape of the surfaces and, mathematically, the slope of the surfaces in the three directions of the 3D space representations are connected to the ability of the models to describe the physicochemical process of the oxy-combustion of the biomass from the point of view of the three factors individually. Although the shapes of the surfaces presented in Figure 4, Figure 5, Figure 6 and Figure 7 were similar, it is necessary to analyse the effect of each factor on the responses separately. This is the only way to determine whether (or not) the trend of each factor explains the chemistry of the oxidation of the biomass and, therefore, to discuss if the optimisation method is valid from a chemical point of view, regardless of the correlation coefficient obtained.

### 3.2. Effects of CO_2_/O_2_ Molar Ratio on the Responses

**Figure 8:**
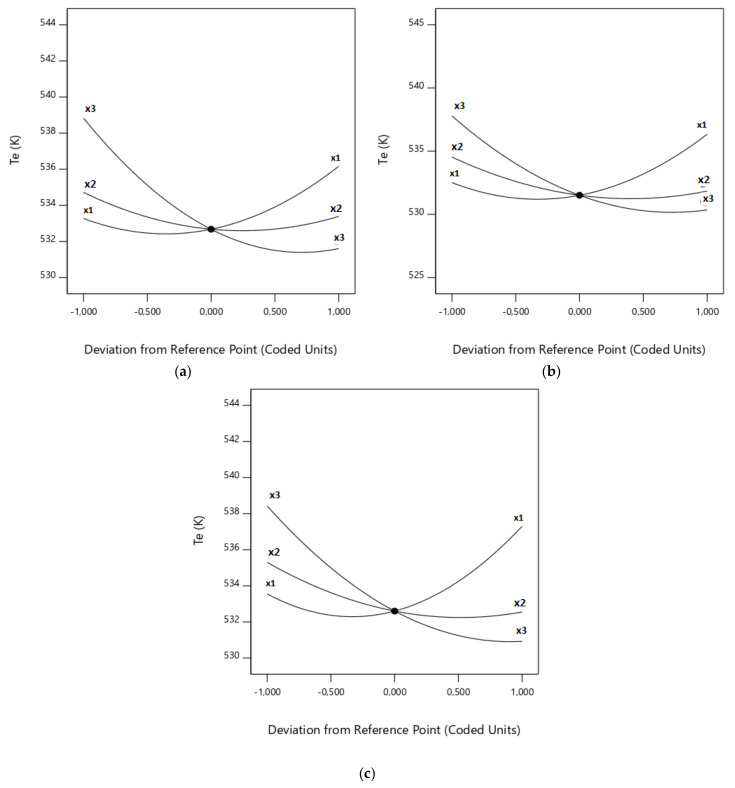
Influence of factors on *T_e_*: (**a**) Complete design; (**b**) Central composite design; and (**c**) Box–Behnken design.

**Figure 9:**
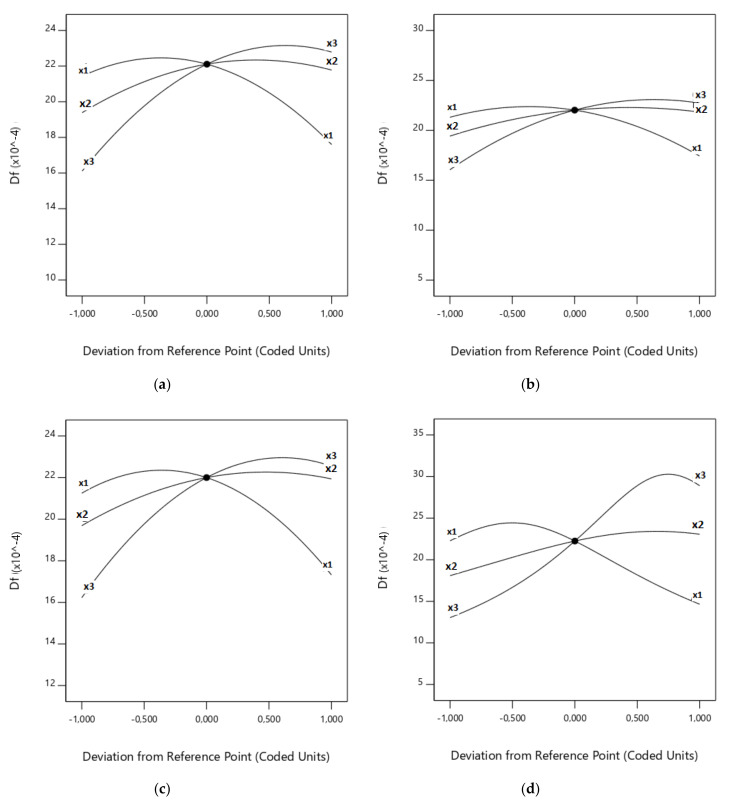
Influence of factors on *D_f_*: (**a**) Complete design; (**b**) Central composite design; (**c**) Box–Behnken design; and (**d**) Central composite design with power transformation.

For closer inspection, the CO_2_/O_2_ molar ratio effects on both responses are presented in Figure 8 and Figure 9. Due to the high sensibility of the responses when the factor varied, it can be concluded that CO_2_/O_2_ molar ratio is an important factor in the oxy-combustion process.

The three main chemical reactions of biomass oxidation are shown in Equations (20–22) [53]:

C + CO_2_ ↔ 2CO; ∆Hr = 1.44 MJ/kg, (20)

C + O_2_ ↔ CO_2_; ∆Hr = −9.20 MJ/kg, (21)

2CO + O_2_ ↔ 2CO_2_; ∆Hr = −12.86 MJ/kg. (22)

Equation (20) represents the CO_2_-biomass and CO_2_-char reactions, where CO_2_ directly reacts with the carbon atoms present in the system. It is a solid–gas reaction. This situation can be found by direct interaction between the virgin biomass and the combustion gases (rich in CO_2_) or by the contact of CO_2_ with the char produced in the combustion of biomass by O_2_. It is an endothermic reaction and, so, its extension is higher at high temperature. On the other hand, Equation (21) represents the oxidation of carbon atoms with O_2_. It is also a solid–gas reaction and can be found in the same situations as Equation (20). However, it is an exothermic reaction. Therefore, its dependence on the temperature is the opposite of that observed in Equation (20). Finally, Equation (22) represents the CO-to-CO_2_ reaction. It is a very exothermic gas–gas reaction which is mainly found in the vicinity of the particle, where the CO concentration is high, due to Equation (20).

Upon closer inspection, Figure 8 and Figure 9 represent how each factor varies between the lowest and highest level proposed in Tables S1–S3 for each response (i.e., *T_e_* in Figure 8 and *D_f_* in Figure 9) and for each RSM model used. For example, considering Figure 9b, it can be seen that an increase in (x_3_) produces an increase in the burnout index, until some value that changes the trend. In the case of the factors (x_1_) and (x_2_), the behaviour can be seen to be the opposite. Furthermore, Figure 8 and Figure 9 allow us to study the slope of the 3D graphs when a single factor is analysed. In these terms, it can be seen that the effect of (x_3_) on *D_f_* is higher than (x_2_) and (x_1_)—in that order—up to a central value (see Tables S1–S3); meanwhile, (x_1_) is the most influential factor on the burnout index of the three factors after the central point, followed by (x_3_) and the (almost horizontal) line that represents (x_2_).

As it can be observed in Figure 8 and Figure 9, there were opposite effects when the CO_2_ concentration was significantly low or high. Therefore, both extremes must be explored. Firstly, as the CO_2_ concentration increases, it is necessary to supply more heat to the system to reach the same particle temperature than that observed when a lower CO_2_ amount is used, due to the higher absorbance of CO_2_ (triatomic) than that of O_2_ (diatomic) [55], as revealed by the higher heat capacity of CO_2_ [56]. As a consequence, as CO_2_ concentration rises, the particle temperature decreases and, so, the ignition temperature of the biomass rises and the burnout index becomes lower. In addition, the higher heat capacity of CO_2_ than that of O_2_ is more significant at moderate oxygen mole fractions (<30%), producing a reduction of the increase of the gas phase temperature induced by the exothermic oxidation of both gas products and char [56]. On the other hand, the endothermic char gasification reactions tend to reduce the maximum temperature of the particle. This is the reason the slope of the CO_2_ graph increases with CO_2_ concentration (*T_e_* response) or decreases (*D_f_* response). This effect can be observed in the three response surface models used in this study.

We can also consider the effect at high temperatures (in the range of 840–1000 K), where the gasification of biomass takes place due to CO_2_. This process is described by Equation (20). As gasification is endothermic, it is favoured at high temperatures. It is also true that an increase of the CO_2_ concentration improves the gasification of biomass (Equation (20)), causing particles to oxidise at lower temperatures. The higher importance of Reaction (Equation (20)) also increases the burnout index. On the other hand, the char can also be oxidised due to O_2_ at lower temperatures, considering Equation (20).

This exothermic reaction is favoured at low–medium temperatures and produces CO_2_ inside the particle channels. Thus, the temperature rises and Reaction (Equation (20)) is favoured. However, if the CO_2_ concentration is low enough, Reaction (Equation (20)) may not be significant. On the other hand, Reaction (Equation (20)) occurs in parallel to the ether bond breakage of the cellulose, hemicellulose, and lignin, which inhibits the exothermic effect that improves Reaction Equation (20)). As a result, although there is a minimum value of CO_2_ flow necessary for reactivity, a relative high concentration of CO_2_ can increase the ignition temperature and decrease the burnout index.

The controversy about the importance of external diffusion in TG processes has not been solved. However, our previous results indicated the significance of external diffusion in the oxy-combustion of a corn–rape blend with the same particle diameter [47]. In addition, Benedetti et al. [57] studied the fluid dynamics of air in the same thermobalance and with the same total flow as those of this research, and found that the Reynolds number was associated with a laminar fluid regime, in the average velocity field around and inside the crucible where reaction occurs, at 0.0126 m/s. As a consequence, it has been suggested that the boundary layer can be characterised as having significative temperature and concentration profiles [58].

Some authors have studied the CO-to-CO_2_ reaction, Equation (21), in TG systems, and found it to be located in the boundary layer [11,59]. Thus, if the CO_2_ concentration rises, the CO_2_ profile in the boundary layer could be reduced, enhancing Reaction (Equation (20)) and producing a persistent CO cloud around the particle. This atmosphere could make the particle burning properties less favourable than that observed at a lower CO_2_ concentrations, due to the higher difficulty of oxygen in accessing the particle surface [20]. In this case, the ignition temperature may be higher and the burnout index lower. Of course, a higher CO_2_ concentration improves Reaction (Equation (20)), which is endothermic, but also reduces the extent of Reaction (Equation (21)), which is exothermic. In both cases, it contributes to reducing the temperature of the particle. As a result, if the CO_2_ concentration is low enough, the CO-to-CO_2_ reaction (exothermic) can be mainly found near the particle surface, reducing the ignition temperature and increasing the burnout index [60].

Finally, Zajdlík et al. [61] deduced that a high CO_2_ concentration decreases the thickness of the ash shell formed during oxidation. This layer is not sufficiently thermally conductive, producing a lower overheating of the particle. In this sense, the ignition temperature calculated from the experiments would increase and the burnout index would decrease.

### 3.3. Effects of the Total Flow on the Responses

The effects of the total flow value are shown in Figure 8 and Figure 9. Due to the high sensitivity of the responses when this factor varies, it can be concluded that this is an important factor in the oxy-combustion process.

As was expected, the total flow value presented two different effects on the values of the responses and, hence, on the oxy-combustion optimisation. On one hand, a very low value of the total flow implies very low values of CO_2_ and O_2_ flows, making the other factors affecting the oxidisation more severe. As a result, a reduction of the total flow increases the ignition temperature and produces a decrease in the burnout index. However, if a higher total flow value is selected, the turbulence outside the particle reduces the range of the temperature profile from the particle surface to the bulk gas. As a result, the heat transmission from the oven to the particle surface and, consequently, across the ash layer over the particle could be higher. It can thus be suggested that the temperature of the surface of the particle could increase, resulting in a decrease in the ignition temperature and an increase in the burnout index. However, in contrast, the elevation of the surface temperature could enhance the endothermic Reaction (Equation (20)), overcooling the particle [11] but making the CO-to-CO_2_ reaction unfavourable. Figure 8 and Figure 9 show that, as a global effect, this latter effect is not important enough, as the ignition temperature decreased and the burnout index increased.

Nevertheless, when a very high total flow is selected, the heat flow between the oven and the particle can change direction and go from the particle surface to the surrounding gases, reducing the temperature of the particle. As a consequence, the ignition temperature increased and the burnout index decreased.

### 3.4. Effects of the Proportion of Rape in the Corn–Rape Blend on the Responses

The effects of the proportion of rape in the corn–rape blend on the responses are shown in Figure 8 and Figure 9. As in the case of the other two factors, the optimal value of the third factor was calculated with the desirability function.

Regarding the ignition parameters presented in previous studies [45], it can be concluded that the ignition sequence is rape > corn, such that it is expected that a high content of rape in the corn–rape blend favours oxidation reactions. This is because the rape possesses a lower carbon content and a higher volatile matter content than corn. Therefore, its oxy-combustion is highly favoured, despite the intermediate content of cellulose and lignin in its structure [45]. Bioresidues with high cellulose and volatile matter content are more favourable in oxy-combustion than bioresidues with high lignin and carbon content. As a result, a high content of rape in the corn–rape blend produces a reduction in the ignition temperature and an increase of the burnout index.

Nevertheless, some authors have pointed out that the burnout index depends on the bioresidue reactivity and char production [45,55]. Following this argument, as carbon content increases, the reactivity of char is improved and the burnout index is also higher. On the other hand, char particles are characterised by a small porosity, which favours the lower diffusion of CO_2_ inside the particle. This facilitates the accumulation of CO_2_ in the mesopores and, so, O_2_ does not reach the particle core. As a result, (see Table 1), as the proportion of rape in the corn–rape blend increases, the oxy-combustion burnout time goes up, the burnout index reduces, and the ignition temperature increases. However, this expected trend was not in accordance with other observations reported in previous works [46] or with the experimental results here reported, which can be resumed as follows: (1) the higher difficulty of CO_2_ accessing the micropores and tortuous channels inside the particle than O_2_ may produce an increase of combustion by O_2_ in these zones. The differences in concentration and temperature profiles cause rape to be more reactive. (2) Holocellulose content (hemicellulose and cellulose) improves reactivity. In addition, in accordance with Jankovic et al. [59], an increase in the volatile matter content produces a higher flame in the particle, producing overheating of the particle. In another work, Zajdlík et al. [61] pointed out that the removal of volatile matter has an influence on the accessibility of the micropore region, enhancing the exothermic reactions of C with O_2_ and causing the particle to become overheated. As a result, (see Table 1), as the rape proportion in the corn–rape blend increases, the ignition temperature decreases and the burnout index increases.

### 3.5. Effects of the Interactions of the Factors on the Responses

The ANOVA results of the mathematical models are listed in Table 3. The study of the interactions between the three factors was completed by a *p*-value analysis of each member of the equation of the corresponding model.

In addition to the data presented in Table 3, Table 4 shows the regression coefficients obtained from the different RSM optimisation models used in this study.

From the regression coefficients listed in Table 4, it can be concluded that the complete design presented the best accuracy of all RSM models. In addition, the complete design can be established as the best study to detect the interactions of the factors here studied. In this design, interactions between factors x_1_ (CO_2_/O_2_ molar ratio) and x_2_ (total flow), factors (x_1_) and x_3_ (% rape in blend), and factors (x_2_) and (x_3_) were found to be significant (*p* < 0.05).

**Figure 10:**
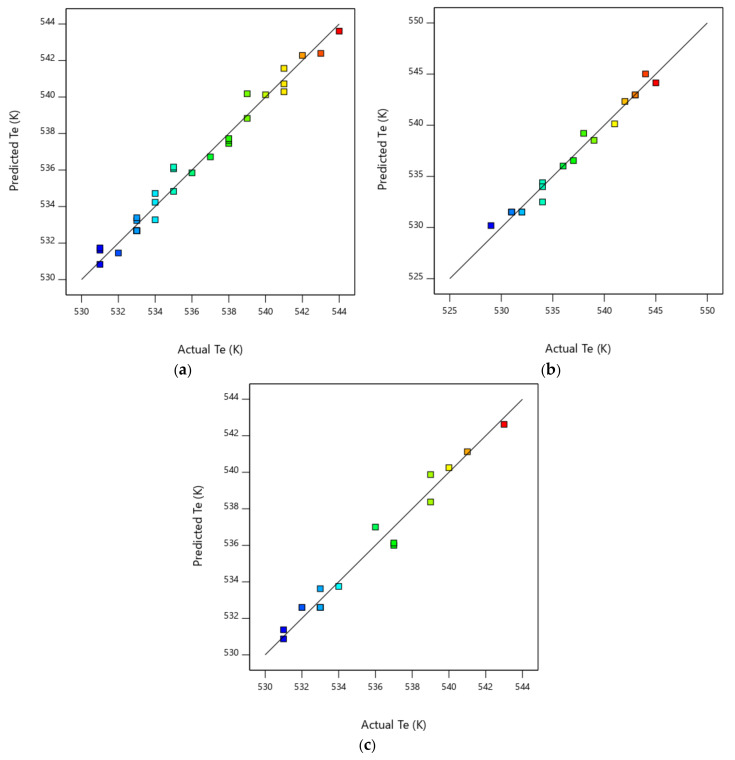
*T_e_* predicted vs. *T_e_* actual: (**a**) Complete design; (**b**) Central composite design; and (**c**) Box–Behnken design.

**Figure 11:**
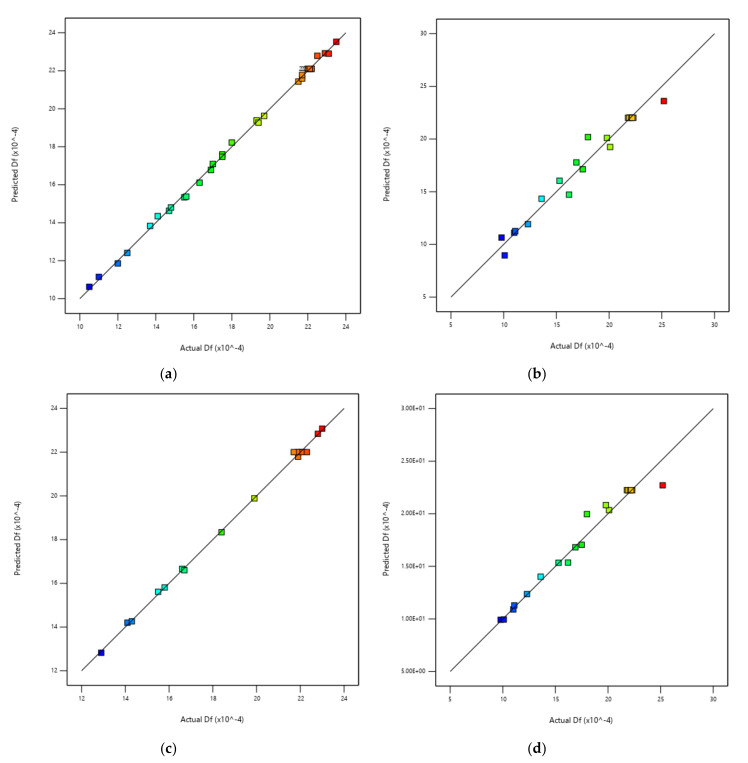
*D_f_* predicted vs. *D_f_* actual: (**a**) Complete design; (**b**) Central composite design; (**c**) Box–Behnken design; and (**d**) Central composite design with power transformation.

The Box–Behnken design predicted no interactions between the three factors for *T_e_* response, while the same interactions that were previously detected in the complete design were observed for the *D_f_* response. However, the accuracy of the *T_e_* prediction (R² predicted = 0.659) was not in reasonable agreement with the regression of the experimental results (Pearson correlation coefficient, R² = 0.974), as the difference was higher than 0.2. This effect can also be seen in Figure 10 and Figure 11, where the predicted vs. actual values are presented. Furthermore, it is true that similar prediction-adjusted regression values were obtained for the *D_f_* response. As a result, the Box–Behnken model did not present a good behaviour, either for data adjustment or data prediction. This conclusion was obtained even when the response results were adjusted by a power transformation.

The central composite design did not predict any interactions between the factors in the case of the *T_e_* response. However, in contrast to the Box-–Behnken results, the similarity of R² pred. and R² was acceptable. In the *D_f_* case, no interactions were detected but the model adjustment was outside the 0.2 admissible limit. This was the reason a power data transformation was performed. Once the data were transformed, the results obtained yielded a very good accuracy; however, a significant interaction between factors x_1_ (CO_2_/O_2_ molar ratio) and x_3_ (% rape in blend) was detected. As a result, the central composite design could be used for data prediction, even though this model only detected one pair factor interaction. A closer inspection allowed for the detection of an outlier value at *D_f_* = 25.0 × 10^−4^ (in both central composite designs). These outliers were obtained when the lowest value of factor x_1_ (CO_2_/O_2_ molar ratio) and the highest value of factor x_3_ (% rape in blend) were used together.

The three paired factor interactions detected in the complete design must be justified from a physico-chemical point of view. In this sense, the coded equation is useful for the identification of the relative impacts of the factors by comparing their coefficients. Table 3 shows that the interaction of factor x_1_ (CO_2_/O_2_ molar ratio) with factor x_3_ (% rape in blend) was found to be the most important. As described by Dhahak et al. [60], lignin oxidation can lead to the formation of anisole and guaiacol, while holocellulose (the mixture of cellulose and hemicellulose) is the source of hydroxyacetaldehyde, 5-methylfurfural, furfural, and furan and its derivatives. When these compounds are oxidised after devolatilisation, anisole and guaiacol can be found to be the highest CO_2_ producers, due to the carbon composition. Therefore, when the proportion of rape in the biomass blend increases, the lignin content decreases and, consequently, the CO_2_ production in the particle decreases. This situation affects the CO_2_-char and CO-to-CO_2_ oxidation reactions. In addition, Adomeit et al. [62] showed that a higher humidity content in the feedstock can enhance the rate of the gas-phase reaction (CO-to-CO_2_), as water vapor can be regarded as an inert species acting as a catalyst. The presence of a higher rape content also increases the moisture content of the blend, affecting the CO-to-CO_2_ reaction in a higher way. Furthermore, higher CO_2_ production also enhances the CO_2_-char reaction, thus affecting the relative amount of CO-CO_2_ in the boundary layer of the particle [57,59].

Factors x_1_ (CO_2_/O_2_ molar ratio) and x_2_ (total flow) can be correlated, as a high total flow can have a significant role in the amount of CO_2_ available inside the oxidation chamber. In addition, a high total flow contributes to an increase in the gas turbulence in the vicinity of the particle. This turbulence can move the CO-to-CO_2_ reaction position, affecting Reactions (Equations (20) and (21)).

Finally, the interaction between factors x_2_ (total flow) and x_3_ (% rape in blend) can be understood once the interactions of factors (x_1_) and (x_3_) and factors (x_1_) with (x_2_) have been explained (by applying the transitive property of group theory).

### 3.6. Comparison of the Relative Impact of the Effects of Factors and Their Interactions on the Responses

**Figure 12:**
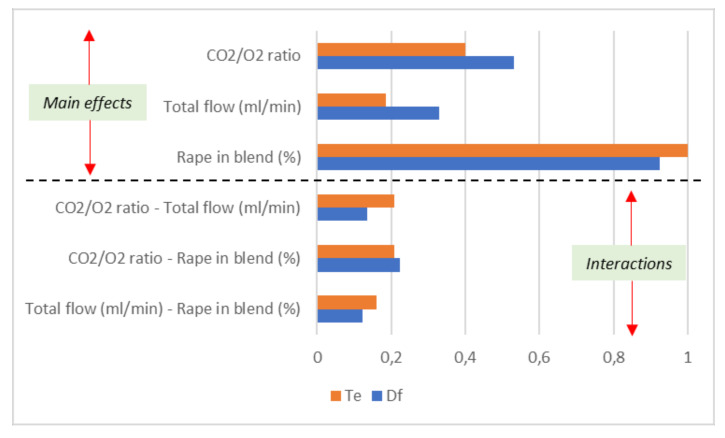
Normalised comparative diagram of the main effects and their interactions in the complete design model for *T_e_* and *D_f_* responses.

From the data shown in Table 4 and from the previous discussions, it can be concluded that, between the RSM, the complete design can be considered the best optimisation method. For this reason, and for the sake of comparison, a normalised comparative diagram of the main effects and their interactions in both responses is presented in Figure 12. Consequently, the results of the coded factor coefficients of “Model 1: complete design”, shown in Table 3, were used as the data set.

Figure 12 is divided into two sections: the main effects and the interactions of factors. The variable with the highest impact on both responses was the proportion of rape in the blend. In other words, the most important variable in the system is the relative composition of cellulose, hemicellulose, and lignin of the blend. This effect is more representative in the *T_e_* response than in *D_f_*. However, the CO_2_/O_2_ ratio and total flow are more important in the *D_f_* results than in the *T_e_* values, probably due to the importance of the CO_2_-char reaction, which directly affects the *D_f_* results. In contrast to the differences shown in the main effects, the interactions of the factors on the responses were similar. Between them, the interaction between the CO_2_/O_2_ ratio and the proportion of rape in the blend in the case of the burnout index was revealed to be the most important, probably also due to the fact that the oxidation of the char by CO_2_ at the end of the process creates a positive synergy between the amount of CO_2_ around the particle and the char formed during the oxidation of the biomass. As a result, the most important variable to be controlled in the optimisation of the oxy-combustion of the blend was the proportion of rape in the blend, followed by the CO_2_/O_2_ ratio present in the combustor.

### 3.7. Application of the ANN as an Optimisation Method

**Figure 13:**
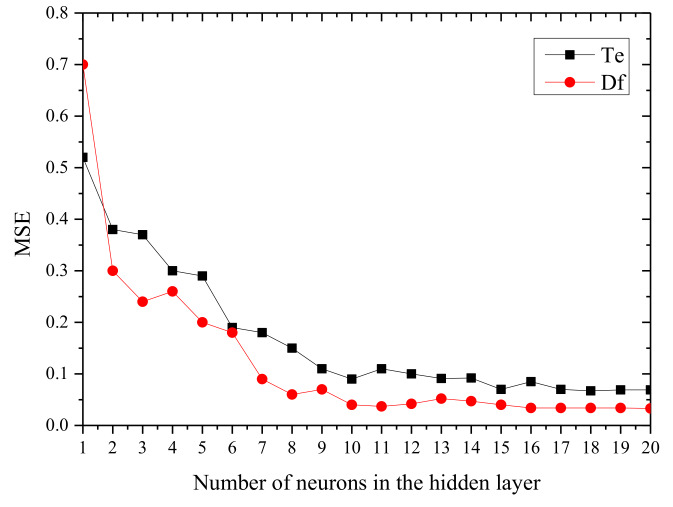
Mean square error (MSE) of a three-layer neural network with different neurons in the hidden layer. (Black colour: ignition temperature. Red colour: burnout index).

The first step in the application of the ANN was to optimise the architecture of the network. This requires knowing the number of hidden layers and the number of neurons in each hidden layer. The first topology tested was [3-*n*-1], where *n* is the number of neurons of the hidden layer. The MSE of this architecture is shown in Figure 13.

It can be seen that, for this configuration, the learning performance first increased and then stabilised with an increase in the number of neurons. This trend was similar to that reported by other authors [27,28]. The MSE stabilised at 0.067 for *T_e_* with 18 neurons and 0.034 for *D_f_* with 16 neurons. To try to reduce the MSE, a four-layer ANN was tested, with the architecture [3-*m*-*n*-1], where *m* and *n* are the numbers of neurons in each hidden layer, respectively. In this case, the optimum MSE found was 0.053 for *T_e_* and 0.012 for *D_f_*, both with an ANN model with 9 neurons in the first hidden layer and 7 neurons in the second hidden layer. It was also noted that the increased number of neurons in the first hidden layer was more helpful in improving the learning performance than that obtained for the second hidden layer. Three and four hidden layers were also computed (tested but not shown here) but it was observed that, while the performance improved slightly and tended to be stable, the networks were more complex. Consequently, the ANN topology was set to the configuration [3,9,7,1] for both ANN_1_ and ANN_2_.

**Figure 14:**
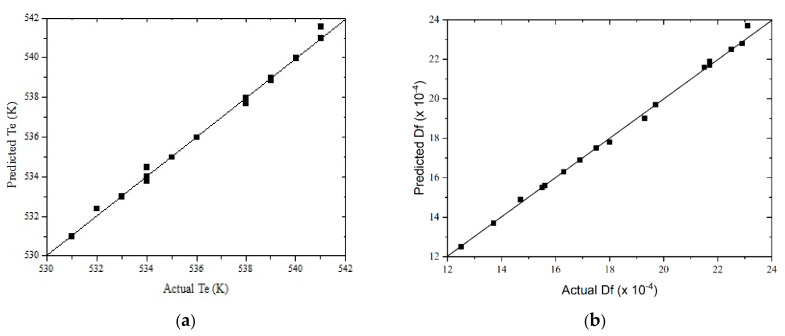
Predicted vs. experimental results of the neural network for: (**a**) *T_e_*, and (**b**) *D_f_*.

The training data set is listed in Table S4 of the Supplementary Materials and, as an example, the results of the training data set for *T_e_* are listed in Table S5. Both weights and biases were satisfactorily computed, and a very high regression coefficient was obtained (see Table 5). Once the ANN_1_ and ANN_2_ parameters were obtained, the models were validated. The experimental conditions used as the validation data set are shown in Table S6. The results for validation of *T_e_* and *D_f_* variables are shown in Figure 14. A very good performance, when comparing predicted and actual variables, was obtained.

Comparing the regression coefficients shown in Table 5 with those obtained for the RSM methods (Table 4), it can be concluded that the ANN method was the best method, with respect not only to the data adjustment but also to data prediction of both responses. The same conclusion can be obtained by comparing Figure 10 and Figure 11 with Figure 14.

### 3.8. Comparison of the Oxy-Combustion Optimisation between RSM and ANN Models

In RSM, the coupled minimisation of the ignition temperature and maximisation of the burnout index requires obtaining the optimal values of the factors at which the optimal values of both responses are obtained. The optimal values that were found in the three RSM methods and the ANN model are listed in Table 6.

In all cases, the results of the optimal values of the factors yielded an intermediate value between the lowest and the highest levels of the three factors. This means that the interval of the values of the factors was properly selected. If the accuracy of the models is observed (Table 4 and Table 5), the complete design can be selected as the best model in terms of the factors and the interactions of the factors. In terms of optimal point predictions, the ANN was very close to the complete design model. These optimal points were an ignition temperature of 531 K and a burnout index of 24.0 × 10^−4^ for the complete design and 533 K and 23.8 × 10^−4^ in the ANN case.

If only the optimal predictions are observed, both the Box–Behnken and central composite designs could be selected as valid optimisation methods. However, the central composite design was observed not to be good enough for regressing the observed–predicted *D_f_* values or to explain the factor interactions. In the case of Box–Behnken, the interactions of the factors for the *T_e_* response could not be explained. The fact of obtaining a good optimal prediction, but not a good individual factor trend explanation, could be justified by error cancellation. As a result, both the Box–Behnken and central composite designs were adequate for the prediction of the optimal oxidation conditions, but not for an adequate explanation of the factors trends. For this reason, these methods cannot be selected as valid mathematical models for the study of biomass oxy-fuel combustion.

When the *D_f_* results with a power data transformation are observed, it can be concluded that the accuracy of the model was notably improved; however, the interactions of the factors could not be described properly. In addition, the optimal value of *D_f_* was far from that obtained with the other models; in particular, from the ANN model and the complete design (which must be established as the most adequate mathematical model of the considered RSM methods). As a result, the power transformation solution for improving the *D_f_* results was not found to be adequate to obtain the optimal operation conditions of the process.

**Figure 15:**
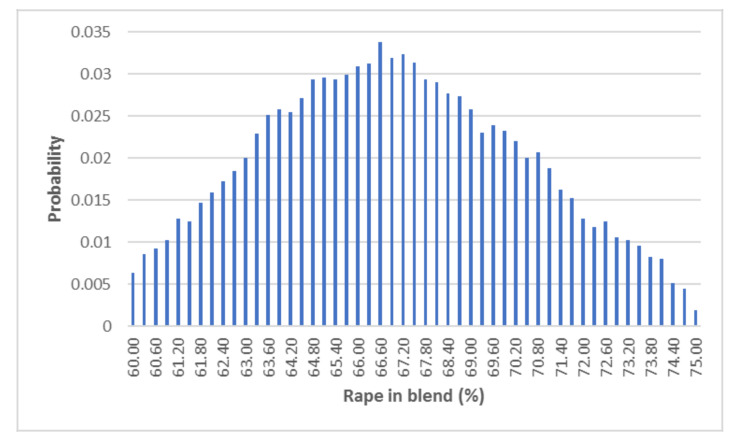
Monte Carlo representation of the complete design optimisation.

To validate the complete design results (and its comparison with the ANN results), the optimal points previously predicted were compared with those obtained by a Monte Carlo simulation. This method has been successfully used in previous oxy-combustion studies [14]. The results, adjusted to a normalised Gaussian curve, are presented in Figure 15.

If the CO_2_/O_2_ molar ratio was maintained at 3.1 and the total flow at 111 mL/min, the expected optimal proportion of rape in the blend was 66.0% ± 3.7%. This result was in accordance with that obtained with the complete design model. As a result, that model was set to be the only one that allows us to obtain a good accuracy, a correct description of the factor behaviours (including their interactions), and a good optimal points prediction.

Finally, the ANN results were compared with the RSM results. When the relative error of RSM results were compared with ANN results, it was concluded that, of the three RSM methods, the complete design presented a deviation from the results of the ANN which were below 1% for both responses. In both cases, the ANN responses were predicted by excess. However, the reduced designs were in agreement with the ANN results only for *T_e_*; while, for *D_f_*, the deviation was higher (double or more than that observed for *T_e_*). The central composite design with power transformation was found to be very far from the ANN results, as well as from the other RSM methods. As a result, the RSM and ANN results were found to be consistent, as a whole, only for the complete design model.

In addition, considering Table 5 and Table 6, the ANN was found to be the best optimisation method of all those applied in this work. From the training data set, the regression coefficient was 0.999 for the *T_e_* and *D_f_* variables, in accordance with the results of other works [23,24,27,28]. A closer inspection allows us to observe that, although the prediction of the *D_f_* variable was not improved, compared with the adjustment provided by the RSM complete design (0.997 in both cases), the regression coefficient increased from 0.955 to 0.996 in the case of the *T_e_* variable. Hence, the ANN was the best optimisation method, in terms of the quality of the regression between all those tested in this study.

As a result, of the previous discussion, the optimal experimental conditions to oxidise a corn–rape blend depended on the method used, but the ANN was proven to be the best adjusted method with the lowest number of experiments needed. Therefore, following this discussion, the point of best operability of the system could be set to a CO_2_/O_2_ molar ratio of 3.3, a total flow of 108 mL/min, and 61% of rape in the biomass blend (ANN optimisation results).

## 4. Conclusions

The conclusions are divided into two categories: the main new findings and those complementary to the findings of other studies.

- Main new findings:

1. The ANN method with two hidden layers is a better optimising method than RSM for oxy-combustion of lignocellulosic biomass. In addition, the ANN needed only 20 experiments to reach the best predictions. For the RSM methods, the most similar model was the complete design model, which required 32 experiments in the training stage. Hence, the ANN was found to be the most efficient model, in terms of good predictions and lower resource needs. The optimum point was found to be a CO_2_/O_2_ molar ratio of 3.3, total flow of 108 mL/min, and 61% of rape in the biomass blend.
2. The complete design model allowed us to successfully discuss the factor behaviours (including all factors interactions) with good accuracy. However, the Box–Behnken and central composite designs only allowed us to discuss the single-factor effects, but not their interactions (even when a power data transformation was used). However, the optimisation of the process was successful for both reduced methods.
3. Of the RSM models, only the complete design should be chosen to 1) study the effects of the CO_2_/O_2_ molar ratio, the total flow, and the proportion of rape in the corn–rape blend on the ignition temperature and the burnout index; 2) study the interactions between the previous factors; and 3) optimise the process in terms of the ignition temperature and the burnout index.

- Findings complementary to other studies:

1. The most important factor to control in the oxy-fuel combustion of the feedstock is the proportion of rape in the blend, where the influence of the factors on the responses can be listed as proportion of rape in blend > CO_2_/O_2_ molar ratio > total flow.

## Figures and Tables

**Table 1:** Proximate analysis, ultimate analysis, calorific value, and composition of principal components corresponding to the corn and rape used in this study.

Material	Corn	Rape
Proximate analysis		
Moisture (%)	8.0	9.2
Volatile matter ^a^ (%)	76.8	80.4
Ash ^a^ (%)	5.7	2.6
Fixed carbon ^a,c^ (%)	17.5	17.0
Ultimate analysis		
C ^b^ (%)	48.8	49.7
H ^b^ (%)	6.2	6.3
N ^b^ (%)	0.5	0.2
S ^b^ (%)	0.1	0.2
O ^b,c^ (%)	44.4	43.6
Calorific value		
HHV (MJ/kg)	18.45	19.49
Composition		
Cellulose (%)	18.6	25.7
Hemicellulose (%)	29.7	21.6
Lignin (%)	12.0	7.4

HHV = high heating value; ^a^ Dry basis; ^b^ Dry ash free basis; ^c^ Calculated by difference.

**Table 2:** Main characteristics of the three response surface methodology (RSM) methods used for optimisation in this study.

RSM Method	Characteristics	Symbol
Complete design	It is the most precise method of the three compared. Rotatable. High number of experiments with more than three factors. In the case of three factors, 32 experiments are needed. Each factor has three levels (−1, 0, 1).	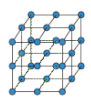
Central composite design	It is a reduced method of the complete design model: for three factors, 19 experiments are needed. Rotatable. Region of operatibility must be greater than region of interest due to axial runs. Each factor has five levels (−α, −1, 0, 1, α).	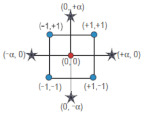
Box–Behnken design	It is a reduced method of the complete design model: for three factors, 17 experiments are needed. Rotatable (or nearly rotatable). Region of operatibility and region of interest nearly the same because there are no axial points. There are no points in the cubic vertex. This leads to worse predictions at the corners of the design space. Each factor has three levels (−1, 0, 1).	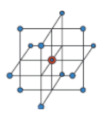

**Table 3:** ANOVA results: effects of the variables on the responses for the complete design, the Box–Behnken design, and the central composite designs.

Factors	y_1_: *T_e_* (K)			y_2_: *D_f_* (× 10^−4^)		
	Uncoded Factors Coefficient	Coded Factors Coefficient	*p*-Values	Uncoded Factors Coefficient	Coded Factors Coefficient	*p*-Values
**Model 1: Complete design**						
Constant	+588.35	+532.68	<0.0001 ^a^	−38.83	+22.11	<0.0001 ^a^
x_1_	−8.39	+1.44	<0.0001 ^a^	+9.17	−1.92	<0.0001 ^a^
x_2_	−0.49	−0.67	0.0002 ^a^	+0.55	+1.19	<0.0001 ^a^
x_3_	−0.53	−3.61	<0.0001 ^a^	+0.56	+3.34	<0.0001 ^a^
x_1_x_2_	+0.02	+0.75	0.0006 ^a^	−0.01	−0.49	<0.0001 ^a^
x_1_x_3_	+0.02	+0.75	0.0006 ^a^	−0.02	−0.81	<0.0001 ^a^
x_2_x_3_	−9 × 10^−4^	−0.58	0.0050 ^a^	+7 × 10^−4^	+0.44	<0.0001 ^a^
x_1_^2^	+0.91	+2.04	<0.0001 ^a^	−1.15	−2.59	<0.0001 ^a^
x_2_^2^	+2.2 × 10^−3^	+1.38	<0.0001 ^a^	−2.4× 10^−3^	−1.52	<0.0001 ^a^
x_3_^2^	+4.1 × 10^−3^	+2.54	<0.0001 ^a^	−4.3 × 10^−3^	−2.66	<0.0001 ^a^
**Model 2: Box–Behnken design**						
Constant	+586.11	+532.60	<0.0001 ^a^	−37.21	+22.00	<0.0001 ^a^
x_1_	−7.54	+1.88	0.0007 ^a^	+10.43	−1.96	<0.0001 ^a^
x_2_	−0.46	−1.37	0.0039 ^a^	+0.45	+1.13	<0.0001 ^a^
x_3_	−0.53	−3.75	<0.0001 ^a^	+0.61	+3.16	<0.0001 ^a^
x_1_x_2_	−6.7 × 10^−3^	−0.25	0.6044	−0.02	−0.60	0.0005 ^a^
x_1_x_3_	+0.01	+0.50	0.3140	−0.03	−1.28	<0.0001 ^a^
x_2_x_3_	+3 × 10^−18^	+0.0	1.0000	+6 × 10^−4^	+0.35	0.0090 ^a^
x_1_^2^	+1.26	+2.82	0.0004 ^a^	−1.21	−2.71	<0.0001 ^a^
x_2_^2^	+2.1 × 10^−3^	+1.32	0.0214 ^a^	−1.9 × 10^−3^	−1.19	<0.0001 ^a^
x_3_^2^	+3.3 × 10^−3^	+2.08	0.0024 ^a^	−4.2 × 10^−3^	−2.61	<0.0001 ^a^
**Model 3: Central composite design**						
Constant	+594.70	+531.51	<0.0001 ^a^	−36.68	+22.02	<0.0001 ^a^
x_1_	−9.46	+1.91	<0.0001 ^a^	+9.34	−1.94	0.0001 ^a^
x_2_	−0.57	−1.35	0.0005 ^a^	+0.50	+1.21	0.0043 ^a^
x_3_	−0.55	−3.72	<0.0001 ^a^	+0.55	+3.34	<0.0001 ^a^
x_1_x_2_	+6.7 × 10^−3^	+0.25	0.4918	−0.01	−0.49	0.2818
x_1_x_3_	+0.02	+0.75	0.0580	−0.02	−0.81	0.0872
x_2_x_3_	−8 × 10^−4^	−0.50	0.1840	+8 × 10^−4^	+0.49	0.2818
x_1_^2^	+1.30	+2.92	<0.0001 ^a^	−1.18	−2.65	<0.0001 ^a^
x_2_^2^	+2.7 × 10^−3^	+1.68	<0.0001 ^a^	−2.2 × 10^−3^	−1.40	0.0014 ^a^
x_3_^2^	+4.1 × 10^−3^	+2.56	<0.0001 ^a^	−4.2 × 10^−3^	−2.63	<0.0001 ^a^
**Model 4: Central composite design with power transformation**						
Constant	Not necessary for *T_e_* response			+0.02	+1.6 × 10^−3^	<0.0001 ^a^
x_1_				−1.9 × 10^−3^	+1.1 × 10^−3^	<0.0001 ^a^
x_2_				−1 × 10^−4^	−5 × 10^−4^	<0.0001 ^a^
x_3_				−3 × 10^−4^	−1.9 × 10^−3^	<0.0001 ^a^
x_1_x_2_				−2 × 10^−6^	−1 × 10^−4^	0.4873
x_1_x_3_				−1 × 10^−5^	−4 × 10^−4^	0.0020 ^a^
x_2_x_3_				+3 × 10^−7^	+2 × 10^−4^	0.0541
x_1_^2^				+5 × 10^−4^	+1.1 × 10^−3^	<0.0001 ^a^
x_2_^2^				+6 × 10^−7^	+4 × 10^−4^	0.0006 ^a^
x_3_^2^				+2 × 10^−6^	+1.2 × 10^−3^	<0.0001 ^a^

^a^ Statistically significant (*p* < 0.05).

**Table 4:** Regression coefficients of the different RSM optimisation models.

Model	*T_e_*		*D_f_*	
	R^2^	R^2^ Predicted	R^2^	R^2^ Predicted
Complete design	0.979	0.955	0.999	0.997
Box–Behnken design	0.974	0.659	0.999	0.993
Central composite design	0.980	0.863	0.967	0.744
Central composite design with power transformation	N/A *	N/A *	0.993	0.944

* N/A: not available.

**Table 5:** Regression coefficients of the artificial neural network models.

Model	*T_e_*		*D_f_*	
	R^2^	R^2^ Predicted	R^2^	R^2^ Predicted
Artificial neural network	0.999	0.996	0.999	0.997

**Table 6:** *T_e_* and *D_f_* values at the optimal point conditions, as found by the five optimisation models.

Model	x_1_: CO_2_/O_2_ Ratio	x_2_: Total Flow (mL/min*)*	x_3_: % Rape in Blend	Optimal Values	Differences with ANN
Artificial neural network	3.3	108	61	*T_e_* (K): 533	-
				*D_f_* (× 10^−4^): 23.8	
Complete design	3.1	111	65	*T_e_* (K): 531	−0.37%
				*D_f_* (× 10^−4^): 24.0	−0.84%
Box–Behnken design	2.6	118	63	*T_e_* (K): 531	−0.37%
				*D_f_* (× 10^−4^): 24.1	1.26%
Central composite design	2.7	115	70	*T_e_* (K): 529	−0.75%
				*D_f_* (× 10^−4^): 24.2	1.68%
Central composite design with power transformation	2.8	114	71	*T_e_* (K): N/A *	N/A *
				*D_f_* (× 10^−4^): 31.6	32.77%

* N/A: not available.

## Supplementary Materials

The following are available online at https://www.mdpi.com/2218-273X/10/5/787/s1, Table S1: Combination of factor values (coded and real levels) in the complete design model; Table S2: Combination of factor values (coded and real levels) in the Box–Behnken model; Table S3: Combination of factor values (coded and real levels) in the central composite models; Table S4: Training data set used in the ANN method; Table S5: The weight and the bias vectors of the neural network model for the evolution of *T_e_*; Table S6: Validation data set used in ANN method.
