# Supplementary material for: A Comparison between Several Response Surface Methodology Designs and a Neural Network Model to Optimise the Oxidation Conditions of a Lignocellulosic Blend
Source: Biomolecules. 2020 May 19;10(5):787. doi: 10.3390/biom10050787 (PMC7277586; doi:10.3390/biom10050787)
Supplement: Supplementary file 1 [file biomolecules-10-00787-s001.pdf]

biomolecules

SUPPLEMENTARY MATERIAL

A comparison between several response surface methodology designs and a neural network model to optimize the oxidation conditions of a lignocellulosic blend

R. López*, C. Fernández, F. J. Pereira, A. Díez, J. Cara, O. Martínez, M.E. Sánchez

Table S1. Combination of factors values (coded and real levels) in the complete design model.

Run	*X_1: CO_2/O_2 relation*		*X_2: Total flow (ml/min)*		*X_3: % rape in blend*	
	Coded	Real	Coded	Real	Coded	Real
**Model 1: Complete design**						
1	-1	2	-1	75	-1	25
2	-1	2	-1	75	0	50
3	-1	2	-1	75	+1	75
4	-1	2	0	100	-1	25
5	-1	2	0	100	0	50
6	-1	2	0	100	+1	75
7	-1	2	+1	125	-1	25
8	-1	2	+1	125	0	50
9	-1	2	+1	125	+1	75
10	0	3.5	-1	75	-1	25
11	0	3.5	-1	75	0	50
12	0	3.5	-1	75	+1	75
13	0	3.5	0	100	-1	25
14	0	3.5	0	100	0	50
15	0	3.5	0	100	+1	75
16	0	3.5	+1	125	-1	25
17	0	3.5	+1	125	0	50
18	0	3.5	+1	125	+1	75
19	+1	5	-1	75	-1	25
20	+1	5	-1	75	0	50
21	+1	5	-1	75	+1	75
22	+1	5	0	100	-1	25

Run	X_1: CO_2/O_2 relation		X_2: Total flow (ml/min)		X_3: % rape in blend	
	Coded	Real	Coded	Real	Coded	Real
23	+1	5	0	100	0	50
24	+1	5	0	100	+1	75
25	+1	5	+1	125	-1	25
26	+1	5	+1	125	0	50
27	+1	5	+1	125	+1	75
28	0	3.5	0	100	0	50
29	0	3.5	0	100	0	50
30	0	3.5	0	100	0	50
31	0	3.5	0	100	0	50
32	0	3.5	0	100	0	50

Table S2. Combination of factors values (coded and real levels) in the Box-Behnken model.

Run	X_1: CO_2/O_2 relation		X_2: Total flow (ml/min)		X_3: % rape in blend	
	Coded	Real	Coded	Real	Coded	Real
**Model 2: Box-Behnken design**						
1	-1	2	-1	75	0	50
2	-1	2	0	100	+1	75
3	-1	2	0	100	-1	25
4	-1	2	+1	125	0	50
5	0	3.5	-1	75	-1	25
6	0	3.5	-1	75	+1	75
7	0	3.5	+1	125	-1	25
8	0	3.5	+1	125	+1	75
9	+1	5	-1	75	0	50
10	+1	5	0	100	-1	25
11	+1	5	0	100	+1	75
12	+1	5	+1	125	0	50
13	0	3.5	0	100	0	50
14	0	3.5	0	100	0	50
15	0	3.5	0	100	0	50
16	0	3.5	0	100	0	50
17	0	3.5	0	100	0	50

biomolecules

MDPI

Table S3. Combination of factors values (coded and real levels) in the central composite models.

Run	*X_1: CO_2/O_2 relation*		*X_2: Total flow (ml/min)*		*X_3: % rape in blend*	
	Coded	Real	Coded	Real	Coded	Real
**Models 3 and 4: Central composite designs**						
1	-1	2	-1	75	-1	25
2	-1	2	-1	75	+1	75
3	-1	2	+1	125	-1	25
4	-1	2	+1	125	+1	75
5	+1	5	-1	75	-1	25
6	+1	5	-1	75	+1	75
7	+1	5	+1	125	-1	25
8	+1	5	+1	125	+1	75
9	-1.682	1	0	100	0	50
10	+1.682	6	0	100	0	50
11	0	3.5	-1.682	58	0	50
12	0	3.5	+1.682	142	0	50
13	0	3.5	0	100	-1.682	8
14	0	3.5	0	100	+1.682	93
15	0	3.5	0	100	0	50
16	0	3.5	0	100	0	50
17	0	3.5	0	100	0	50
18	0	3.5	0	100	0	50
19	0	3.5	0	100	0	50

Table S4. Training data set used in ANN method.

Run	*X_1: CO_2/O_2 relation*		*X_2: Total flow (ml/min)*		*X_3: % rape in blend*	
	Coded	Real	Coded	Real	Coded	Real
1	-1.682	1	-1	25	0	50
2	-1.682	1	0	100	0	50
3	-1.682	1	0	125	+1	75
4	-1	2	-1	75	-1	25
5	-1	2	-1	75	+1	75
6	-1	2	+1	125	-1	25
7	-1	2	+1	125	+1	75
8	0	3.5	-1.682	58	-1	25

Run	X_1: CO_2/O_2 relation		X_2: Total flow (ml/min)		X_3: % rape in blend	
	Coded	Real	Coded	Real	Coded	Real
9	0	3.5	-1.682	58	0	50
10	0	3.5	0	100	-1.682	8
11	0	3.5	0	100	0	50
12	0	3.5	0	100	+1.682	93
13	0	3.5	+1.682	142	0	50
14	0	3.5	+1.682	142	+1	75
15	+1	5	-1	75	+1	75
16	+1	5	+1	125	-1	25
17	+1	5	-1	75	-1	25
18	+1	5	+1	125	+1	75
19	+1.682	6	0	100	0	50
20	+1.682	6	+1	125	0	50

Table S5. The weight and the biases vectors of the neural network model for the evolution of T_e.

**i/j**	**1**	**2**	**3**	**4**	**5**	**6**	**7**	**8**	**9**
		w_{ij}							
1	-0.953	2.358	1.259	-0.478	-0.184	3.587	2.618	-0.186	-2.845
2	3.687	-2.241	-0.615	1.510	0.145	0.678	-3.561	-0.948	-4.164
3	0.184	-0.684	-3.867	4.386	0.816	0.279	-2.681	-1.768	0.181
		θ_j							
	1.761	-0.046	0.167	0.617	-0.351	-0.164	-2.345	4.315	2.674
**j/k**	**1**	**2**	**3**	**4**	**5**	**6**	**7**		
		w'_{jk}							
1	-1.641	0.150	0.861	0.186	-3.186	-1.168	-0.984		
2	2.186	-1.482	-2.546	0.784	0.478	0.187	3.187		
3	0.175	-1.048	-0.546	2.684	1.974	-0.468	-0.873		
4	3.843	-2.731	0.413	1.764	1.375	-0.387	-0.189		
5	-4.518	0.678	0.178	0.162	-1.468	-0.468	0.479		
6	0.041	0.164	-0.087	-0.016	1.471	2.451	3.784		
7	0.495	0.490	-1.694	-0.048	-0.098	1.894	1.186		
8	-0.689	-0.984	0.311	0.025	-0.297	-0.009	-0.014		
9	-3.483	-0.698	1.687	-0.267	-3.597	1.110	0.154		
		θ'_k							
	0.468	3.468	-0.046	-0.871	2.674	0.364	-0.478		

k/1	1
	w’’$_k$
1	4.316
2	-0.486
3	3.468
4	-0.021
5	3.468
6	-2.475
7	-0.268
	θ’’
	-0.111

Table S6. Validation data set used in ANN method.

Run	*X_1: CO_2/O_2 relation*		*X_2: Total flow (ml/min)*		*X_3: % rape in blend*	
	Coded	Real	Coded	Real	Coded	Real
1	-1	2	-1	75	0	50
2	-1	2	0	100	-1	25
3	-1	2	0	100	0	50
4	-1	2	0	100	+1	75
5	-1	2	+1	125	0	50
6	0	3.5	-1	75	-1	25
7	0	3.5	-1	75	0	50
8	0	3.5	-1	75	+1	75
9	0	3.5	0	100	-1	25
10	0	3.5	0	100	+1	75
11	0	3.5	+1	125	-1	25
12	0	3.5	+1	125	0	50
13	0	3.5	+1	125	+1	75
14	+1	5	-1	75	0	50
15	+1	5	0	100	-1	25
16	+1	5	0	100	0	50
17	+1	5	0	100	+1	75
18	+1	5	+1	125	0	50
